# Prevalence of obstructive sleep apnea syndrome and predictors of difficult orotracheal intubation

**DOI:** 10.1590/1806-9282.20240347

**Published:** 2024-09-16

**Authors:** Jefferson Thiago Medeiros de Oliveira, Heitor Medeiros, Wallace Andrino

**Affiliations:** 1 Onofre Lopes University Hospital, Anesthesiology Service – Natal (RN), Brazil.; 2 Massachusetts General Hospital, Department of Anesthesiology, Critical Care and Pain Medicine – Boston (MA), United States.

**Keywords:** Apnea, Airway obstruction, Anesthesia, Intraoperative complication, Prevalence

## Abstract

**OBJECTIVE:**

Obstructive sleep apnea syndrome (OSAS) is an underdiagnosed condition that causes recurrent episodes of partial or total collapse of the upper airways during sleep. It is associated with perioperative pulmonary complications. The STOP-BANG is a screening tool for assessing patients at risk of OSAS. The aim of this study was to assess the prevalence of patients with OSAS during pre-anesthetic evaluation at a hospital and its correlation with predictors of ventilation and difficult airway.

**METHODS:**

This is an observational, cross-sectional study carried out from January 2022 to September 2023. The questionnaire comprised demographic data (age, weight, BMI, type of surgery, and anesthesia), the STOP-BANG, predictors of difficult orotracheal intubation (Mallampati, mouth opening, thyromental distance, cervical mobility, and upper lip bite test), and predictors of difficult ventilation through a facial mask (male sex, absence of teeth, presence of beard, obesity, and >55 years).

**RESULTS:**

The study had the participation of 221 patients, of whom 121 presented with a STOP-BANG ≥3, with a prevalence of 54.2%. All patients undergoing bariatric surgeries presented STOP-BANG ≥3. No significant statistical relationships were found between predictors of difficult orotracheal intubation and STOP-BANG ≥3. However, significant statistical relationships were found in relation to predictors of difficult ventilation through the facial mask.

## INTRODUCTION

Obstructive sleep apnea syndrome (OSAS) is a disease characterized by recurrent episodes of partial or total collapse of the upper airways during sleep, resulting in reduced or absent airflow for at least 10 s and, consequently, oxygen desaturation and nocturnal awakenings^1^. Maintaining upper airway patency is of great concern in patients with obstructive sleep apnea due to a higher predisposition to changes in the tone of the pharyngeal muscles during sleep, especially under anesthesia, a circumstance in which the depressive effect of the drugs used for sedation has the additional capacity to reduce the tone of this musculature^2^.

The routine use of screening tools in the preoperative period is important to identify patients with undiagnosed OSAS. Many tools for screening patients with OSAS have been proposed, such as the Berlin questionnaire, the STOP-BANG questionnaire, and the American Society of Anesthesiologists (ASA) classification, and their use increases the likelihood of identifying OSAS preoperatively^3^.

The STOP-BANG questionnaire is an easy-to-administer tool that consists of eight questions, as designated by the acronym STOP-BANG (Snoring, Tiredness during daytime, Observed apnea, high blood pressure, Body mass index, Age, Neck circumference, Gender)^1^. Some studies have shown that patients with OSAS present difficult intubation in 13–24% of cases, with the need for awake intubation in 8%^4^.

The objectives of this study are to assess the prevalence of patients with OSAS during pre-anesthetic evaluation at Onofre Lopes University Hospital and to correlate with the predictors of difficult orotracheal intubation and difficult ventilation under face mask.

## METHODS

This study was approved by the Ethics and Research Committee of the Onofre Lopes University Hospital (CAAE: 52664321.2.0000.5292). It is an observational, cross-sectional study, with data collection from January 2022 to September 2023, about 1 year and 9 months, evaluating the prevalence of patients with STOP-BANG ≥3 undergoing elective surgeries under general anesthesia, as well as the correlation with predictors of difficult intubation and difficult facial mask ventilation.

The patients underwent pre-anesthetic evaluations at an outpatient level or in the ward for patients already admitted, who were not evaluated in the anesthesiology outpatient clinic of HUOL. The study aimed to analyze all eligible patients collected within the designated timeframe, reflecting a comprehensive and practical overview of the clinical scenarios encountered.

### Inclusion criteria

-Patients admitted to HUOL for elective surgery under general anesthesia and who were evaluated in a pre-anesthetic consultation were included in the study.

### Exclusion criteria

-Patient refusal, patients under 18 years of age, and patients with predictors of difficult airway with planned use of a fibroscope for difficult orotracheal intubation were excluded from the study.

A questionnaire consisting of demographic data (age, weight, BMI, type of surgery, and anesthesia), STOP-BANG as a screening tool, five predictors of difficult orotracheal intubation (Mallampati, mouth opening, thyromental distance, cervical mobility, upper lip bite test), and the five predictors of difficult ventilation under a facial mask (male sex, absence of teeth, presence of beard, obesity, >55 years) was applied. To measure neck circumference and thyromental distance, a measuring tape was used.

### Statistical analysis

The chi-square and Fisher's exact tests were used to examine the relationship between the groups of categorical variables. To identify potential factors associated with the studied outcomes (STOP-BANG ≥3, STOP-BANG<3), a significance level of α=0.05 was used. Additionally, standardized residuals were analyzed to identify statistically different groups. The analyses were performed using the R language (R Core Team, 2020).

The sample size calculation was performed using the G*Power 3.1.9.4 software, considering a significance level of α=0.05, test power of 80%, and an effect size of w=0.30, resulting in a sample size of 160 patients.

## RESULTS

The study involved the participation of 221 patients, of whom 120 presented with STOP-BANG ≥3, with a prevalence of 54.2%. A larger number of patients than the calculated size was collected to enhance the power of the tests and reduce effect measures. Considering the same effect size used in the previous sample size calculation (w=0.30), a power of 0.92 was found. We performed a chi-square test to analyze the relationship between surgery type and STOP-BANG ≥3, revealing significant differences.

The chi-square test assumptions required that no more than 20% of expected frequencies be below five and none be zero. We combined categories with fewer than 10 observations into an "others" category to meet these criteria. Standardized residual analysis identified significant differences. Residuals with an absolute value ≥1.96 (α=0.05) were considered significant, indicating that the observed value differs statistically from the expected value if surgery type and STOP-BANG ≥3 were independent. Positive residuals indicate observed values higher than expected, while negative residuals indicate lower values. Significant differences were found for CCP and bariatric surgery: CCP was more related to STOP-BANG<3, and bariatric surgery was more related to STOP-BANG ≥3 (Table 1).

**Table 1:** Hypothesis test for the type of surgery according to STOP-BANG ≥3.

			STOP-BANG ≥3		p-value
			No (n=101)	Yes (n=120)	
**Type of surgery**					0.026
	General/digestive	50 (23%)	23 (-0.24)	27 (0.24)	
	ENT	32 (14%)	18 (1.07)	14 (-1.07)	
	Urological	43 (19%)	15 (-1.85)	28 (1.85)	
	CCP	17 (7.7%)	12 (1.98)	5 (-1.98)	
	Neurosurgery	16 (7.2%)	9 (0.73)	7 (-0.73)	
	Bariatric	15 (6.8%)	2 (-2.75)	13 (2.75)	
	Coloproctolóica	14 (6.3%)	7 (0.19)	7 (-0.19)	
	Other	34 (15%)	19 (1.06)	15 (-1.06)	
p<0.05; Pearson's chi-squared test (standardized residuals)					

This table presents the chi-square test results examining the relationship between surgery types and STOP-BANG scores (≥3 or <3). Significant differences were observed, with bariatric surgery being more associated with STOP-BANG ≥3, and CCP more associated with STOP-BANG <3. Standardized residuals are shown in parentheses, with values ≥1.96 indicating statistical significance (p<0.05).

No statistically significant relationships were found between predictors of difficult orotracheal intubation and a STOP-BANG score ≥3 (Table 2).

**Table 2:** Hypothesis tests for predictors of difficult orotracheal intubation according to STOP-BANG ≥3.

			STOP-BANG ≥3		p-value
			No (n=101)	Yes (n=120)	
Mouth opening <3 cm	No	180 (81%)	79 (78%)	101 (84%)	0.299
	Yes	41 (19%)	22 (22%)	19 (16%)	
Mallampati >2	No	146 (66%)	72 (71%)	74 (62%)	0.155
	Yes	75 (34%)	29 (29%)	46 (38%)	
Cervical mobility	No	15 (6.8%)	5 (5%)	10 (8.3%)	0.424
	Yes	206 (93%)	96 (95%)	110 (92%)	
Thyromental distance <6 cm	No	194 (89%)	87 (86%)	109 (91%)	0.293
	Yes	25 (11%)	14 (14%)	11 (9.2%)	
Upper lip test >grade II	No	194 (88%)	89 (88%)	105 (88%)	>0.999
	Yes	27 (12%)	12 (12%)	15 (13%)	

This table shows Fisher's exact test results for predictors of difficult orotracheal intubation in relation to STOP-BANG scores (≥3 or <3). The features evaluated include mouth opening <3 cm, Mallampati score >2, cervical mobility, thyromental distance <6 cm, and upper lip test >grade II. The table presents the distribution of these predictors in both STOP-BANG ≥3 and <3 groups, along with their corresponding p-values.

For a STOP-BANG score ≥3, it was found that there is a statistically significant relationship with the absence of teeth (p-value 0.014), obesity (p-value <0.001), age>55 years (p-value <0.001), and male gender (p-value <0.001) (Table 3).

**Table 3:** Hypothesis testing for the predictors of difficult ventilation under a face mask according to STOP-BANG ≥3.

			STOP-BANG ≥3		p-value
			No (n=101)	Yes (n=120)	
Absence of teeth	No	152 (69%)	78 (77%)	74 (62%)	0.014
	Yes	69 (31%)	23 (23%)	46 (38%)	
Presence of beard	No	201 (91%)	95 (94%)	106 (88%)	0.163
	Yes	20 (9.0%)	6 (5.9%)	14 (12%)	
Obesity	No	150 (68%)	88 (87%)	62 (52%)	<0.001
	Yes	71 (32%)	13 (13%)	58 (48%)	
Age >55 years	No	147 (67%)	80 (79%)	67 (56%)	<0.001
	Yes	74 (33%)	21 (21%)	53 (44%)	
Male	No	126 (57%)	70 (69%)	56 (47%)	<0.001
	Yes	95 (43%)	31 (31%)	64 (53%)	

This table presents Fisher's exact test results for predictors of difficult ventilation under a face mask, categorized by STOP-BANG scores (≥3 or <3). Significant predictors include the absence of teeth, obesity, age >55 years, and male gender (p<0.05). The presence of a beard did not show a significant difference between the groups. The table displays the distribution and percentages of these predictors in both STOP-BANG ≥3 and <3 groups.

## DISCUSSION

The application of the STOP-BANG score is essential for screening elective patients, as it has been validated across a wide range of populations^5^. It has been particularly noted for its effectiveness in screening for OSA in surgical patients, demonstrating high predictive accuracy^6^. The number of patients with OSAS in the general surgery/digestive system and urology groups was higher than those in the bariatric group, indicating that some external factors may have contributed to this result. Grouping general and digestive system patients together may increase the sample size of this group. As for urology, it is considered the surgical specialty with the highest demand for patients in the surgical center of Onofre Lopes University Hospital. Since the questionnaire for this study was applied randomly, not all days of the week were covered uniformly, allowing some specialties to show higher prevalence due to being evaluated more times than others.

Regardless of the surgical specialty, a large portion of patients with OSAS scheduled for elective surgery remain undiagnosed^1,7^. Preoperative screening for OSAS is important for preparing strategies to minimize perioperative risks.

For this, the STOP-BANG questionnaire is useful in predicting difficult intubation, especially in obese patients. Identifying patients with OSAS through this analysis during pre-anesthetic evaluation and being prepared for possible airway management difficulties would help prevent adverse events related to difficult orotracheal intubation and difficult ventilation with a face mask^8^. It was observed that the prevalence of OSAS is particularly high in patients undergoing gastric bypass surgery, showing a strong association between OSAS and obesity^9^.

In this study, we examined five predictors of difficult orotracheal intubation in correlation with a STOP-BANG score of ≥3: <3 cm mouth opening, Mallampati score >2, thyromental distance <6 cm, cervical mobility, and the upper lip bite test. Unlike previous findings^10^, no significant statistical relationships were observed between these predictors and difficult intubation scenarios. It is important to note that the absence of statistical significance in this study does not imply that these predictors are irrelevant for predicting OSAS.

A sample of 221 patients was obtained and showed a high prevalence of OSAS. Subsequent studies emphasize the importance of a good pre-anesthetic evaluation to identify predictors of difficult intubation and reduce the risks associated with a difficult airway due to difficult orotracheal intubation.

In our study, morbidly obese patients with a STOP-BANG score ≥3 demonstrated a significantly increased risk of airway management difficulties. This finding underscores the utility of the STOP-BANG score in predicting challenges with orotracheal intubation within this demographic, as it was previously related in other scenarios^11^. Elevated neck circumference and a Mallampati score of III or IV are also reported as key predictors of difficulty during laryngoscopy^12^. Furthermore, these predictors were confirmed in a subgroup of obese patients with OSAS in another sample^13^.

The evaluation of the five predictors of difficult ventilation under a face mask according to STOP-BANG ≥3 in this work showed a significant statistical relationship between presenting OSAS and having no teeth, obesity, age>55 years, and male sex. The presence of a beard was the only predictor that did not show statistical relevance and differed from most studies. The fact of being in the Northeast region of Brazil, with higher temperatures, and a cultural aspect of men not having voluminous beards, determines that the presence of a beard, specifically its size, is not a determinant in this correlation.

Several factors can predict possible difficulty in ventilation with a face mask, with OSAS being one of them. The STOP-BANG questionnaire ≥3 has a high negative predictive value and can be very useful in ruling out the possibility of difficult ventilation with a mask^14^. In a prospective study, 9 out of 10 patients with difficult mask ventilation at induction suffered from OSAS^15^. In another retrospective study with 1,399 patients, factors predictive of difficult mask ventilation were identified as age >47 years, history of difficult intubation, BMI>35 kg/m^2^, short neck, neck circumference >40 cm, presence of a beard, and history of OSAS. The absence of teeth as a main contributor to difficult ventilation under a face mask was found in another study^8^.

## CONCLUSION

This study confirms a high prevalence of OSAS among elective surgery patients at Onofre Lopes University Hospital, validating the STOP-BANG questionnaire's effectiveness in pre-anesthetic evaluations. However, no significant relationships were found between STOP-BANG scores and predictors of difficult orotracheal intubation. These results highlight the need for targeted strategies to manage ventilation challenges in patients with OSAS.

## ETHICS STATEMENT

The project was previously approved by the Research Ethics Committee of the Onofre Lopes University Hospital (CEP/Huol), CAAE: 52664321.2.0000.5292.

## Supplementary Material


