# Supplementary material for: Prevalence of obstructive sleep apnea syndrome and predictors of difficult orotracheal intubation
Source: Rev Assoc Med Bras (1992). 2024 Sep 16;70(9):e20240347. doi: 10.1590/1806-9282.20240347 (PMC11404991; doi:10.1590/1806-9282.20240347)
Supplement: Supplementary file 1 [file 1806-9282-ramb-70-09-e20240347-supp01.docx]

**PREVALENCE OF OBSTRUCTIVE SLEEP APNEA SYNDROME AND PREDICTORS OF DIFFICULT OROTRACHEAL INTUBATION**

**Sample Size Calculation**

To analyze the power for tests aimed at examining the prevalence of patients with a STOP-BANG score ≥3 undergoing elective surgeries under general anesthesia conditions, as well as the correlation with predictors of difficult intubation and difficult mask ventilation, the sample size calculation was performed considering the following information:

- **Type of Surgery**: 8 categories (General/CAD, otorhinolaryngology, urology, CCP, neurosurgery, bariatric, colorectal, others);
- **STOP-BANG ≥ 3**: 2 categories (Yes, No);
- **Significance level (α)**: 0.05;
- **Statistical test considered**: Chi-square test;
- **Effect size**: The type of effect measure considered in this calculation was Cohen's w. Three scenarios were considered for this calculation:
  - **Effect Size** | **Value considered (w)**
  - Small | 0.10
  - Medium | 0.30
  - Large | 0.50

The software used for the analysis was G*Power 3.1.9.7.

| **Value** | **Sample size** |
| --- | --- |
| 0.10 | 1436 |
| 0.15 | 638 |
| 0.20 | 359 |
| 0.25 | 230 |
| 0.30 | 160 |
| 0.35 | 118 |
| 0.40 | 90 |
| 0.45 | 71 |
| 0.50 | 58 |

Given the established value of 221 patients for the research, a sample size calculation was performed to ensure that this sample size is sufficient to demonstrate the study's objective. The power of the test was calculated for different effect sizes as shown in the table below:

| **Effect size** | **Power of the test** |
| --- | --- |
| 0.10 | 0.15 |
| 0.15 | 0.31 |
| 0.20 | 0.55 |
| 0.25 | 0.78 |
| 0.30 | 0.92 |
| 0.35 | 0.98 |
| 0.40 | 0.99 |
| 0.45 | 0.99 |
| 0.50 | 0.99 |
